# Oncological and functional outcomes in T3 and T4 laryngeal cancer patients: choice for larynx preservation or total laryngectomy based on expected laryngeal function

**DOI:** 10.1017/S0022215124000112

**Published:** 2024-06

**Authors:** Arman Nobacht, Tineke W H Meijer, Sjoukje F Oosting, Bert van der Vegt, Jan Wedman, György B Halmos, Boudewijn E C Plaat

**Affiliations:** 1 Department of Otorhinolaryngology, Head and Neck Surgery, University Medical Center Groningen, University of Groningen, Groningen, The Netherlands; 2 Department of Radiotherapy, University Medical Center Groningen, University of Groningen, Groningen, The Netherlands; 3 Department of Medical Oncology, University of Medical Center Groningen, University of Groningen, Groningen, The Netherlands; 4 Department of Pathology and Medical Biology, University Medical Center Groningen, University of Groningen, Groningen, The Netherlands

**Keywords:** Laryngeal squamous cell carcinoma, radiotherapy, chemoradiotherapy, laryngectomy

## Abstract

**Objective:**

To determine oncological and functional outcomes in patients with T3 and T4 laryngeal carcinoma, in which choice of treatment was based on expected laryngeal function and not T classification.

**Methods:**

Oncological outcomes (disease-specific survival and overall survival) as well as functional outcomes (larynx preservation and functional larynx preservation) were analysed.

**Results:**

In 130 T3 and 59 T4 patients, there was no difference in disease-specific survival or overall survival rates after radiotherapy (RT) (107 patients), chemoradiotherapy (36 patients) and total laryngectomy (46 patients). The five-year disease-specific survival rates were 83 per cent after RT, 78 per cent after chemoradiotherapy and 69 per cent after total laryngectomy, whereas overall survival rates were 62, 54 and 60 per cent, respectively. Five-year larynx preservation and functional larynx preservation rates were comparable for RT (79 and 66 per cent, respectively) and chemoradiotherapy (86 and 62 per cent, respectively).

**Conclusion:**

There is no difference in oncological outcome after (chemo)radiotherapy or total laryngectomy in T3 and T4 laryngeal carcinoma patients whose choice of treatment was based on expected laryngeal function.

## Introduction

Since the introduction of organ preservation for advanced laryngeal cancer, it has been debated which primary treatment modality, larynx preserving (chemo)radiotherapy (i.e. radiotherapy with or without chemotherapy) or larynx sacrificing surgery, results in a superior clinical outcome.^1–4^ Studies showed that patients with locally advanced laryngeal carcinomas, staged as T3 or T4, can be treated with (chemo)radiotherapy to preserve the larynx without compromising survival.^5^ However, patients who present with impaired swallowing and/or airway obstruction, and in whom a functional larynx after treatment is not expected by a multidisciplinary team (MDT), total laryngectomy should be performed.^5^ In contrast to these studies, the current National Comprehensive Cancer Network guidelines advises treating all T4a tumours with total laryngectomy.^6^

In our institution, the choice of primary treatment for T3 or T4 laryngeal cancer is based on MDT advice, which considers N classification and the estimated functionality of the larynx after definitive (chemo)radiotherapy. It should be emphasised that it is the expected laryngeal function, rather than the T classification, that is critical in the decision to refer a patient for laryngeal preservation treatment or laryngectomy. Currently, there is a lack of recent data about oncological and functional outcomes in laryngeal cancer patients treated with such an approach.

Most studies of the optimal treatment strategy for advanced laryngeal cancer focus on overall survival.^7^ However, laryngeal cancer patients are prone to co-morbidities and other malignancies, therefore disease-specific survival provides better insights regarding oncological outcome after treatment for laryngeal cancer.^8^ Functional outcomes after larynx preserving treatment are often evaluated by anatomical larynx preservation. However, functional larynx preservation would provide better indirect insight into quality of life over time.^9^ The combined data of disease-specific survival and functional larynx preservation of a recently treated cohort could reveal possible progress in the treatment of advanced laryngeal cancer.

This retrospective study aimed to provide updated laryngeal cancer treatment results by investigating both oncological and functional outcomes of patients with a T3 or T4 laryngeal squamous cell carcinoma, diagnosed and treated between 2010 and 2018, and recently treated by (chemo)radiotherapy or total laryngectomy. The choice of treatment was based on an MDT meeting that estimated laryngeal function after definitive (chemo)radiotherapy.

## Methods

### Patients

This retrospective cohort contained 200 consecutive patients treated with curative intent for a T3 or T4 laryngeal squamous cell carcinoma in a single tertiary referral centre at the Head and Neck Cancer Centre of the University Medical Centre Groningen between 2010 and 2018. Follow up continued until March 2021.

All patients were discussed in an MDT meeting (involving dedicated head and neck oncologic surgeons, radiation oncologists, medical oncologists, pathologists, (nuclear medicine) radiologists, nurses and dentists) to select the treatment of choice, based on estimated laryngeal function definitive (chemo)radiotherapy. The estimated post-treatment laryngeal function was based on signs of aspiration, dysphagia, stridor and tumour involvement in the laryngeal skeleton before treatment. Aspiration was assessed by swallowing water with or without methylene blue, or, in cases of doubt, swallowing and aspiration were evaluated by videofluorography. However, T3 or T4 classification itself did not determine the choice of treatment.

The advice of the MDT was extensively discussed with each patient and his/her family by the head and neck oncological surgeon, a dedicated nurse and a speech pathologist, and additional information regarding chemotherapy and radiotherapy was provided by radiation and medical oncologists. Most patients received additional (comprehensive) geriatric assessment. Patients could give informed consent at least a week after receiving all the information.

Eleven patients were excluded for the following reasons: died before treatment (one patient), died during radiotherapy (four patients), received cetuximab instead of chemotherapy (four patients), could not undergo post-operative radiotherapy (one patient) or lost to follow up (one patient).

The following variables were registered: age, sex, date of diagnosis, date of first treatment, last date of follow up, tumour–node–metastasis staging according to the seventh edition of the American Joint Committee on Cancer Staging Manual (2009), treatment characteristics, follow-up status, tumour location, American Society of Anaesthesiologists classification, laryngeal function (i.e. tracheostomy and/or feeding tube dependency at date of last follow up) and, if applicable, salvage surgery.

Based on Dutch medical research law (Wet Medisch-Wetenschappelijk Onderzoek met mensen), our Institutional Review Board concluded that this retrospective study (202200055) fulfilled all the requirements and was in accordance with the regulations.

### Treatment

Patients received standardised treatment regimens, but some variation within this treatment regimen was inevitable. In summary, laryngectomy, with or without additional neck dissection, was performed under general anaesthesia by dedicated and registered head and neck oncological surgeons. Patients who were treated with primary radiotherapy received a total dose of 70 Gy (in 35 fractions of 2.00 Gy) to the primary tumour and pathological lymph nodes. A bilateral elective dose of 35 × 1.55 Gy (total dose of 54.25 Gy) was given to cervical lymph node levels II, III and IV. In the case of pathological lymph nodes, more lymph node levels received an elective dose. Radiotherapy, using an intensity-modulated radiotherapy technique, was prepared by using planning computed tomography, magnetic resonance imaging and/or ^18^F-fluoro-deoxy-glucose positron emission tomography of the head and neck region in radiotherapy positioning.

All patients had an indication for post-operative radiotherapy (with or without chemotherapy) after total laryngectomy. Thirty-three fractions of 2.0 Gy (total dose 66.0 Gy) or 28 fractions of 2.0 Gy (total dose 56.0 Gy) were delivered to the primary tumour, depending on tumour-free margins and the presence of adverse prognostic factors (perineural growth, lymph- and/or angio invasion and spidery growth). An elective dose of 33 fractions of 1.6 Gy (total dose 52.8 Gy) was delivered to cervical lymph node levels II, III and IV in cases of N0 (i.e. without lymph node metastases). In cases of lymph node metastases (N+), more lymph node levels received an elective dose. In the case of lymph node metastases with extra nodal spread, the total dose to these lymph node areas was 33 × 2.0 Gy (total dose 66.0 Gy). Lymph node metastases without extranodal spread were treated with a 56 Gy equivalent dose (i.e. 28 × 2 Gy or 33 × 1.8 Gy).

Chemotherapy consisted of three cycles of cisplatin (100 mg/m^2^) or carboplatin (300–350 mg/m^2^) with 5-fluor-uracil (5-FU; 600 mg/m^2^ as a continuous infusion for 96 hours) in a three-week cycle. Chemotherapy was considered for patients younger than 70 years with nodal involvement or large T3 or T4 tumours. Post-operative chemoradiotherapy was considered for patients with extranodal extension or resections with no clear margins. A speech pathologist treated each patient after total laryngectomy and patients with speaking or swallowing problems after (chemo)radiotherapy.

### Statistical analysis

For statistical analysis, patients were divided into three treatment groups: (1) radiotherapy (RT), (2) chemoradiotherapy or (3) total laryngectomy with post-operative (chemo)radiotherapy. In every survival analysis primary RT was used as the reference category. N classifications were grouped into (1) N0 and (2) N+ (containing N1, N2 and N3). Descriptive statistics were used to describe the patient cohort. Pearson chi-square and the Mann–Whitney *U*-test were used to compare the baseline characteristics of the treatment groups.

The primary endpoints of this study were disease-specific survival and overall survival. Disease-specific survival was defined as the time from start of first therapy until death due to laryngeal cancer or treatment-related death. All other cases were censored at the date of other cause of death or the date of the last follow up. Overall survival was defined as the time from start of first therapy until death from any cause. All other cases were censored at the date of the last follow up.

Secondary endpoints were larynx preservation and functional larynx preservation. Larynx preservation was defined as the time from start of first therapy until total laryngectomy. Functional larynx preservation was defined from start of first therapy until date of local recurrence or total laryngectomy, at any time, or tracheostomy and/or feeding tube dependency, two years after therapy.

The Kaplan–Meier method was used for assessment of survival rates and survival curves. Cox regression was used for univariate and multivariate analysis of disease-specific survival, overall survival, larynx preservation and functional larynx preservation. Univariate statistically significant variables and treatment modalities were included in the multivariate analysis using a significance level of 5 per cent (two-tailed). Statistical analysis was performed using IBM SPSS Statistics 23 for Microsoft Windows (SPSS, Chicago, Illinois, USA) and StataCorp 2009 Stata Statistical Software, Release 11 (StataCorp LP, College Station, Texas, USA). Figures were drawn using GraphPad Prism (v9.3.1, GraphPad Software, La Jolla, California, United States).

## Results

In total, 189 patients were included, of whom 130 (69 per cent) were registered with a T3 tumour and 59 (31 per cent) were registered with a T4 tumour (Table 1). Of all the included patients, 104 (55 per cent) were registered with a supraglottic carcinoma, 71 (37 per cent) with a glottic carcinoma, 5 (3 per cent) with a subglottic carcinoma and 9 (5 per cent) with a transglottic carcinoma. Patient and tumour characteristics are shown in Table 1.

**Table 1.** Patient and tumour characteristics for the treatment regimens RT, CRT and TL, including age, gender, T classification, N classification, American Society of Anaesthesiologists classification, tumour location (i.e. supraglottic, glottic, subglottic or transglottic) and total radiotherapy dose (Gy, post-operative patient usually received 56 or 66 Gy)

	RT (*N* = 107)	CRT (*N* = 36)	TL (*N* = 46)	*p* value
Age (*n* (IQR))	67 (61–73)	61 (57–66)	66 (60–72)	**<0**.**01**
Gender (*n* (%))				0.386
– Male	88 (82)	26 (72)	38 (83)	
– Female	19 (18)	10 (28)	8 (17)	
T classification (*n* (%))				**<0**.**01**
– T3	93 (87)	24 (67)	13 (28)	
– T4	14 (13)	12 (33)	33 (72)	
N classification (*n* (%))				**<0**.**01**
– N0	88 (82)	7 (19)	22 (48)	
– N1	6 (6)	8 (22)	7 (15)	
– N2	13 (12)	21 (58)	15 (33)	
– N3	0 (0)	0 (0)	2 (4)	
– N+	19 (18)	29 (81)	24 (52)	
American Society of Anaesthesiologists classification (*n* (%))				**0**.**01**
– 1	12 (11)	3 (8)	1 (2)	
– 2	59 (55)	29 (80)	26 (57)	
– 3	35 (33)	4 (11)	16 (35)	
– 4	1 (1)	0 (0)	3 (7)	
Tumour location (*n* (%))				**<0**.**01**
– Supraglottic	48 (45)	27 (75)	29 (63)	
– Glottic	54 (51)	7 (19)	10 (22)	
– Subglottic	3 (3)	0 (0)	2 (4)	
– Transglottic	2 (2)	2 (6)	5 (11)	
Total radiotherapy dose (*n* (%))				**<0**.**01**
– 56 Gy	0 (0)	0 (0)	18 (39)	
– 60 Gy	0 (0)	0 (0)	1 (2)	
– 66 Gy	0 (0)	0 (0)	26 (57)	
– 70 Gy	107 (100)	36 (100)	1 (2)	

Values in bold are statistically significant. Abbreviations: RT radiotherapy, CRT chemoradiotherapy, TL total laryngectomy; IQR = interquartile range; Gy Gray

### Treatment regimens

Patients were treated with primary RT in 107 cases (57 per cent), chemoradiation in 36 cases (19 per cent) and total laryngectomy in 46 cases (24 per cent). Ten per cent (13 out of 130) of T3 staged tumours were treated with total laryngectomy, compared with 56 per cent (33 out of 59) of T4 staged tumours (*p* < 0.01).

The treatment groups differed significantly in age, T classification, N classification, tumour location and American Society of Anaesthesiologists classification (Table 1). Patients in the chemoradiation group were younger (median age, 60.6 years) than patients in the RT (median age, 66.8 years) or total laryngectomy (median age, 66.3 years) groups. The total laryngectomy group included more patients with T4 tumours than the RT and chemoradiation groups. The chemoradiation and total laryngectomy groups comprised more patients with lymph node metastasis compared with the RT group. Patients treated by chemoradiotherapy had significantly more American Society of Anaesthesiologists 2 classifications compared with the RT and total laryngectomy groups. The RT-treated group contained more glottic tumours and fewer N+ cases compared with the chemoradiation group or the total laryngectomy groups.

### Disease-specific survival and overall survival

Of the 189 patients, 102 (54 per cent) survived, 37 (19 per cent) died of laryngeal carcinoma, 42 (22 per cent) died of other causes and 8 (4 per cent) were lost to follow up. Of the 8 patients of 70 years or older with N+ disease (i.e. an indication for chemotherapy in younger patients), 6 received radiotherapy without chemotherapy and 3 died of disease. Two of the older patients who were judged fit for chemotherapy had no evidence of disease during follow up (*p* > 0.05). Of the 20 patients younger than 70 years of age, 17 received chemotherapy (4 died of disease) and 3 were judged not fit for chemotherapy, 2 of whom died of disease during follow up (*p* > 0.05).

**Figure 1.**
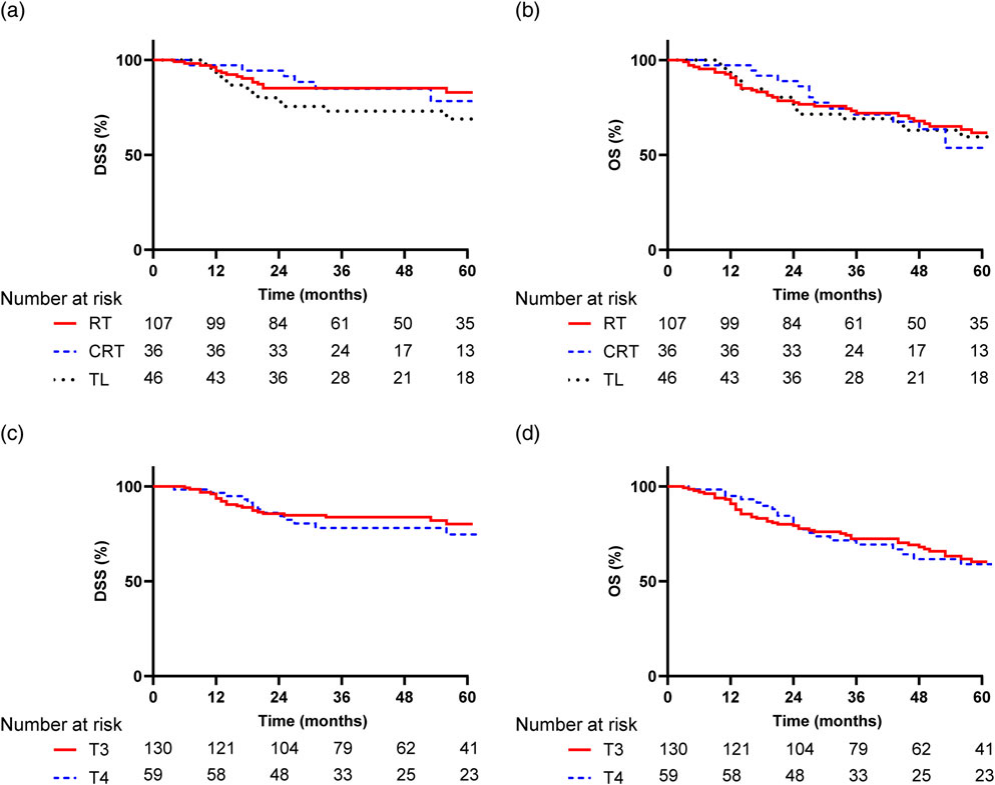
Kaplan–Meier curves for (a) DSS and (b) OS for each treatment regimen and (c) DSS and (d) OS for T3 and T4 staged tumours. DSS = disease-specific survival; RT = radiotherapy; CRT = chemoradiotherapy; TL = total laryngectomy; OS = overall survival

The median follow-up time was 41.9 months (range, 3–124). The 5-year disease-specific survival rate after RT was 83 per cent (95 per cent confidence interval (CI), 83–91 per cent), after chemoradiation was 78 per cent (95 per cent CI, 60–96 per cent) and after total laryngectomy was 69 per cent (95 per cent CI, 55–83 per cent) (Figure 1a). The 5-year overall survival rates were 62 per cent (95 per cent CI, 52–72 per cent) for RT, 54 per cent (95 per cent CI, 34–74 per cent) for chemoradiation and 60 per cent (95 per cent CI, 44–76 per cent) for total laryngectomy (Figure 1b).

There was no significant difference in disease-specific survival (*p* = 0.387) or overall survival (*p* = 0.748) rates between any of the treatment groups. Five years after treatment, the disease-specific survival rate was 80 per cent (95 per cent CI, 72–88 per cent) for T3 and 75 per cent (95 per cent CI, 63–87 per cent) for T4 staged tumours, whereas the 5-year overall survival rate was 60 per cent (95 per cent CI, 50–70 per cent) for T3 and 59 per cent (95 per cent CI, 45–73 per cent) for T4 staged tumours (Figure 1c,d). There was no significant difference in disease-specific survival (*p* = 0.752) or overall survival (*p* = 0.920) rates between patients with T3 or T4 laryngeal carcinomas.

### Larynx preservation and functional larynx preservation

**Figure 2.**
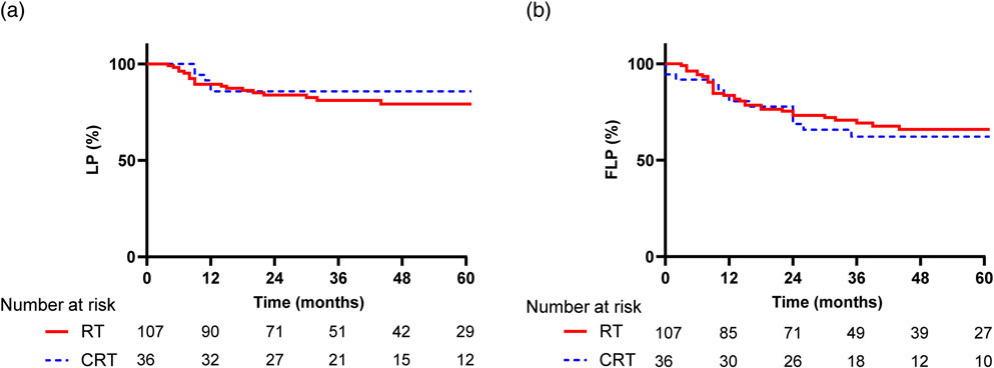
Kaplan–Meier curves for (a) LP and (b) FLP for RT and CRT. LP = larynx preservation; RT = radiotherapy; CRT = chemoradiation; FLP = functional larynx preservation

Out of a total of 189 patients with a T3 or T4 laryngeal carcinoma, larynx preservation was achieved in 62 per cent and functional larynx preservation was achieved in 50 per cent at 5 years after treatment. Of the 143 patients who underwent larynx preserving treatment, the 5-year larynx preservation rate was 81 per cent (95 per cent CI, 73–89 per cent) and the 5-year functional larynx preservation rate was 65 per cent (95 per cent CI, 57–73 per cent). As shown in Figure 2a, the 5-year larynx preservation rates for the RT and chemoradiation groups were 79 per cent (95 per cent CI, 71–87 per cent) and 86 per cent (95 per cent CI, 74–98 per cent), respectively.

Five years after treatment, the functional larynx preservation rate was 66 per cent (95 per cent CI, 56–76 per cent) for RT-treated patients and 62 per cent (95 per cent CI, 46–78 per cent) for chemoradiotherapy-treated patients (Figure 2b). In patients with T3 stage tumours, the 5-year larynx preservation and functional larynx preservation rates were 83 per cent (95 per cent CI, 75–91 per cent) and 66 per cent (95 per cent CI, 56–76 per cent), respectively, and in patients with T4 stage tumours the 5-year larynx preservation and functional larynx preservation rates were 72 per cent (95 per cent CI, 52–92 per cent) and 58 per cent (95 per cent CI, 36–80 per cent), respectively.

### Failure of functional larynx preserving treatment

Failure of functional larynx preservation treatment occurred in 32 out of 107 patients (29.9 per cent) who received RT and in 13 out of 36 patients (36.1 per cent) who received chemoradiation. In 32 out of 45 of these cases (71 per cent), patients had a local recurrence, 8 out of 45 patients (18 per cent) were feeding tube dependent, 10 out of 45 (22 per cent) had a persistent tracheostomy and 3 (7 per cent) had a life-threatening non-functional larynx after RT. Of the 32 patients with a local recurrence, 24 were initially treated with RT (75 per cent) and 8 with chemoradiation (25 per cent). A salvage laryngectomy could be performed in 20 patients. The other 12 patients did not undergo salvage surgery because the tumour was inoperable because of gross tumour extension (*n* = 4) or proven distant metastasis (*n* = 2), the patient died (*n* = 2), the patient decided not to have surgery (*n* = 2) or the patient had a poor condition (*n* = 2).

### Predictors of disease-specific survival and overall survival

Apart from N classification and American Society of Anaesthesiologists classification, no other significant predictors for disease-specific survival and overall survival were identified using univariate analysis. Multivariate analysis showed that the only independent predictive factor for worse disease-specific survival was N+ (*p* = 0.002; Table 2). Patients diagnosed with N+ and patients with an American Society of Anaesthesiologists 4 score had significantly worse overall survival rates, with *p* < 0.001 and *p* < 0.05, respectively (Table 2).

**Table 2.** Multivariate analyses for disease-specific survival and overall survival

Parameter	Disease-specific survival		Overall survival	
	HR (95% CI)	*p* value	HR (95% CI)	*p* value
Treatment				
– Radiotherapy	Reference		Reference	
– Chemoradiotherapy	0.47 (0.16–1.39)	0.12	0.61 (0.30–1.24)	0.168
– Total laryngectomy	0.87 (0.39–1.97)	0.74	0.61 (0.33–1.14)	0.119
ASA classification				
– ASA 1	Reference		Reference	
– ASA 2	1.10 (0.24–5.15)	0.90	1.21 (0.37–4.00)	0.76
– ASA 3	2.01 (0.41–9.88)	0.39	2.61 (0.78–8.77)	0.20
– ASA 4	4.53 (0.52–39.41)	0.17	7.4 (1.31–41.78)	**0**.**023**
N classification				
– N0	Reference		Reference	
– N+	3.24 (1.47–7.32)	**0**.**005**	3.05 (1.70–5.50)	**<0**.**001**

Variables included in the analysis were treatment regimen (i.e. radiotherapy, chemoradiotherapy and total laryngectomy), ASA classification and N classification. HR = hazard ratio; CI = confidence interval; ASA = American Society of Anaesthesiologists

## Discussion

Our study shows that larynx preserving treatment and larynx sacrificing treatment yield high rates of both disease-specific survival and overall survival in T3 or T4 laryngeal cancer patients. In contrast to previous studies, our study shows that oncological outcome did not significantly differ between patients treated with radiotherapy, chemoradiotherapy or total laryngectomy, nor between T3 and T4 laryngeal cancer patients.^7^ More than 80 per cent of the patients treated with (chemo)radiotherapy achieved anatomical larynx preservation, while two-thirds of larynx preservation patients had a functional larynx 5 years after treatment.

Our results suggest that oncological and functional outcomes in patients with laryngeal squamous cell carcinomas may have improved over the past decade. This could be explained by improved treatment regimens and discussing the potential chance of a functional larynx after definitive (chemo)radiotherapy for each patient in an MDT, resulting in better patient selection for larynx preservation. In addition, MDT-guided treatment selection is associated with better survival rates and has been recently recommended.^10–12^ During an MDT meeting, the patient's general condition and comorbidities are taken into account when considering treatment.^11^

In recent decades, multiple studies have focussed on overall survival, demonstrating controversial results.^7^ The majority of the studies showed favourable outcomes for total laryngectomy for T4 laryngeal cancer, but for T3 tumours the choice of treatment did not seem to influence overall survival.^7,13,14^ For the last 10 years, only a few studies have focused on disease-specific survival as the main indicator of oncological outcome.^7^ The study by Lorenzo *et al*. showed similar findings in T3 laryngeal cancer, but detected lower disease-specific survival rates (RT 66.1 per cent and chemoradiation 71.6 per cent) for larynx preservation than our study for T3 and T4 combined (RT 83 per cent and chemoradiation 77 per cent).^15^ In a meta-analysis, also combining T3 and T4 laryngeal carcinomas, total laryngectomy was found to be superior to larynx preservation with regard to disease-specific survival.^7^ However, this meta-analysis is characterised by heterogeneity and 2 out of the 4 included studies were conducted before 2000, therefore they did not include recent improvements in radiotherapy and patient selection for larynx preservation.^3,7,16^

Our study shows that functional larynx preservation rates are substantially lower than larynx preservation rates, therefore using just larynx preservation as a marker for successful therapy would result in overestimation of functional outcomes. As a non-functional larynx negatively affects quality of life, functional larynx preservation is a better readout for functional outcome.^1,9,17^ A nationwide Dutch study showed larynx preservation rates in line with our study, ranging from 77 to 87 per cent, with a trend favouring chemoradiation.^18^ Results from a study by Rosenthal *et al*. showed that larynx preservation in T4 laryngeal cancer patients resulted in 5 years of freedom from local recurrence–tracheostomy–gastrostomy rate of 50 per cent, which is comparable to the 58 per cent for 5-year functional larynx preservation found in the present study.^19^

- There is still a debate whether larynx preserving (chemo)radiotherapy results in superior clinical outcome compared with larynx sacrificing surgery
- Decisions on treatment of T3 and T4 laryngeal carcinoma can also be determined by the expected laryngeal and pharyngeal function after (chemo)radiotherapy
- No differences in disease-specific survival and overall survival were observed after a weighted choice for a larynx preserving or sacrificing treatment based on expected laryngeal function after definitive (chemo)radiotherapy
- The 5-year larynx preservation after (chemo)radiotherapy for T3/T4 laryngeal carcinoma was 80 per cent with a functional larynx in 65 per cent
- Only 19 per cent of the patients died of T3-T4 laryngeal cancer and 54 per cent of the patients were alive after 5 years

According to the institutional practice covered in this study, patients above 70 years of age do not receive chemotherapy, based on the results of the meta-analysis of Pignon *et al*.,^20^ therefore chemoradiation patients are significantly younger and have a lower American Society of Anaesthesiologists classification than patients who only received radiotherapy. Also, T4 stage tumours are more likely to cause laryngeal dysfunction and are therefore treated more often with total laryngectomy. This could result in worse survival rates for total laryngectomy patients compared with chemoradiation patients. Another limitation of this study is the limited number of patients in the total laryngectomy and chemoradiation groups, which meant it was not possible to perform a valid subgroup analysis.

## Conclusion

Current treatment regimens based on estimated laryngeal function after definitive (chemo)radiotherapy, continues to improve disease-specific survival and overall survival rates in T3 or T4 laryngeal cancer patients. No differences in disease-specific survival and overall survival rates were observed between (chemo)radiotherapy and total laryngectomy treatments. Five years after primary (chemo)radiotherapy, two-thirds of patients had a functional larynx.

## References

[1] Forastiere AA, Weber RS, Trotti A. Organ preservation for advanced larynx cancer: issues and outcomes. J Clin Oncol 2015;33:3262–8 26351339 10.1200/JCO.2015.61.2978 PMC5320920

[2] Sanabria A, Chaves ALF, Kowalski LP, Wolf GT, Saba NF, Forastiere AA et al. Organ preservation with chemoradiation in advanced laryngeal cancer: the problem of generalizing results from randomized controlled trials. Auris Nasus Larynx 2017;44:18–25 27397024 10.1016/j.anl.2016.06.005

[3] Chen AY, Fedewa S, Zhu J. Temporal trends in the treatment of early-and advanced-stage laryngeal cancer in the United States, 1985–2007. Arch Otolaryngol Head Neck Surg 2011;137:1017–24 22006780 10.1001/archoto.2011.171

[4] Patel SA, Qureshi MM, Dyer MA, Jalisi S, Grillone G, Truong MT. Comparing surgical and nonsurgical larynx-preserving treatments with total laryngectomy for locally advanced laryngeal cancer. Cancer 2019;125:3367–77 31206637 10.1002/cncr.32292

[5] Forastiere AA, Ismaila N, Lewin JS, Nathan CA, Adelstein DJ, Eisbruch A et al. Use of larynx-preservation strategies in the treatment of laryngeal cancer: American Society of Clinical Oncology clinical practice guideline update. J Clin Oncol 2018;36:1143–69 29172863 10.1200/JCO.2017.75.7385

[6] Haddad RI, Hicks WL, Hitchcock YJ, Jimeno A, Leizman D, Maghami E et al. NCCN Clinical Practice Guidelines in Oncology *(*NCCN Guidelines®*)* for Guideline Head and Neck Cancers, Version 2.2022. © National Comprehensive Cancer Network, Inc., 2022.

[7] Tang ZX, Gong JL, Wang YH, Li ZH, He Y, Liu YX et al. Efficacy comparison between primary total laryngectomy and nonsurgical organ-preservation strategies in treatment of advanced stage laryngeal cancer a meta-analysis. Medicine *(*Baltimore*)* 2018;97:1–6 10.1097/MD.0000000000010625 PMC6392597 29794737

[8] Gao X, Fisher SG, Mohideen N, Emami B. Second primary cancers in patients with laryngeal cancer: a population-based study. Int J Radiat Oncol Biol Phys 2003;56:427–35 12738317 10.1016/s0360-3016(02)04613-8

[9] Murphy BA, Ridner S, Wells N, Dietrich M. Quality of life research in head and neck cancer: a review of the current state of the science. Crit Rev Oncol Hematol 2007;62:251–67 17408963 10.1016/j.critrevonc.2006.07.005

[10] Pan CC, Kung PT, Wang YH, Chang YC, Wang ST, Tsai WC. Effects of multidisciplinary team care on the survival of patients with different stages of non-small cell lung cancer: a national cohort study. PLoS One 2015;10:1–13 10.1371/journal.pone.0126547 PMC4429114 25966317

[11] Friedland PL, Bozic B, Dewar J, Kuan R, Meyer C, Phillips M. Impact of multidisciplinary team management in head and neck cancer patients. Br J Cancer 2011;104:1246–8 21448166 10.1038/bjc.2011.92 PMC3078600

[12] Takes RP, Halmos GB, Ridge JA, Bossi P, Merkx MAW, Rinaldo A et al. Value and quality of care in head and neck oncology. Curr Oncol Rep 2020;22:92 32651680 10.1007/s11912-020-00952-5 PMC7351804

[13] Grover S, Swisher-Mcclure S, Mitra N, Li J, Cohen RB, Ahn PH et al. Total laryngectomy versus larynx preservation for T4a larynx cancer: patterns of care and survival outcomes. Int J Radiat Oncol Biol Phys 2015;92:594–601 26068492 10.1016/j.ijrobp.2015.03.004

[14] Dziegielewski PT, O'Connell DA, Klein M, Fung C, Singh P, Mlynarek MA et al. Primary total laryngectomy versus organ preservation for T3/T4A laryngeal cancer: a population-based analysis of survival. J Otolaryngol Head Neck Surg 2012;41:56–64 22569051

[15] García Lorenzo J, Montoro Martínez V, Rigo Quera A, Codina Aroca A, López Vilas M, Quer Agustí M et al. Modifications in the treatment of advanced laryngeal cancer throughout the last 30 years. Eur Arch Otorhinolaryngol 2017;274:3449–55 28625009 10.1007/s00405-017-4639-z

[16] Dietz A, Wiegand S, Kuhnt T, Wichmann G. Laryngeal preservation approaches: considerations for new selection criteria based on the DELOS-II trial. Front Oncol 2019;9:1–7 31355142 10.3389/fonc.2019.00625 PMC6635549

[17] Terrell JE, Fisher SG, Wolf GT. Long-term quality of life after treatment of laryngeal cancer. Arch Otolaryngol Head Neck Surg 1998;124:964–71 9738804 10.1001/archotol.124.9.964

[18] Timmermans A, van Dijk BAC, Overbeek LIH, van Velthuysen MLF, van Tinteren H, Hilgers FJM et al. Trends in treatment and survival for advanced laryngeal cancer: a 20-year population-based study in the Netherlands. Head Neck 2015;38:1247–55 10.1002/hed.24200 26315454

[19] Rosenthal DI, Mohamed ASR, Weber RS, Garden AS, Sevak PR, Kies MS et al. Long-term outcomes after surgical or nonsurgical initial therapy for patients with T4 squamous cell carcinoma of the larynx: a 3-decade survey. Cancer 2015;121:1608–19 25586197 10.1002/cncr.29241 PMC4424158

[20] Pignon JP, Maître A le, Maillard E, Bourhis J. Meta-analysis of chemotherapy in head and neck cancer (MACH-NC): an update on 93 randomised trials and 17,346 patients. Radiother Oncol 2009;92:4–14 19446902 10.1016/j.radonc.2009.04.014

